# Linguistic signs in action: The neuropragmatics of speech acts

**DOI:** 10.1016/j.bandl.2022.105203

**Published:** 2023-01

**Authors:** Rosario Tomasello

**Affiliations:** Brain Language Laboratory, Department of Philosophy and Humanities, Freie Universität Berlin, 14195 Berlin, Germany; Cluster of Excellence ‘Matters of Activity. Image Space Material’, Humboldt Universität zu Berlin, 10099 Berlin, Germany

**Keywords:** Neuropragmatics, Speech acts, Communicative functions, Pragmatics, Dialogue structure, Social interaction, Language games, Context, EEG, fMRI

## Abstract

What makes human communication exceptional is the ability to grasp speaker’s intentions beyond what is said verbally. How the brain processes communicative functions is one of the central concerns of the neurobiology of language and pragmatics. Linguistic-pragmatic theories define these functions as speech acts, and various pragmatic traits characterise them at the levels of propositional content, action sequence structure, related commitments and social aspects. Here I discuss recent neurocognitive studies, which have shown that the use of identical linguistic signs in conveying different communicative functions elicits distinct and ultra-rapid neural responses. Interestingly, cortical areas show differential involvement underlying various pragmatic features related to theory-of-mind, emotion and action for specific speech acts expressed with the same utterances. Drawing on a neurocognitive model, I posit that understanding speech acts involves the expectation of typical partner follow-up actions and that this predictive knowledge is immediately reflected in mind and brain.

## Highlights

• Speech acts are embedded in actions and settings defining the function they convey.
• These include the action sequence, structure commitments, and other social pragmatic aspects.
• Rapid neural processing of pragmatic features in parallel with semantic information.
• Specific cortical areas reflect the processing of specific pragmatic features.

## 1 Pragmatics and the brain

Language is a communication system that allows us to efficiently express our intentions to others. Yet, the processes by which a listener grasps speaker’s intentions, which often go beyond the uttered expression (Grice, 1957, Levinson, 1983, Wittgenstein, 1953), are still an open matter. This is because there is a many-to-many relationship between the linguistic utterance and the various possible functions it may have in communicative interactions (Ehlich, 2007, Fritz, 2013, Wittgenstein, 1953). For instance, the expression “here is an apple” can be used to teach someone the meaning of a word, to draw attention to a particular object or to offer that object upon request. To capture the pragmatic meaning of a linguistic utterance in social interactions, several processes are at work at the linguistic, contextual, and social levels (Grice, 1975, Levinson, 1983, Noveck and Sperber, 2004). These processes have long been researched in philosophy and linguistics, but only in recent decades has it become a field of research in neuroscience known as “Neuropragmatics” (Bambini et al., 2011, Bara et al., 1997, Cutica et al., 2006, Gambi et al., 2015, Hagoort and Levinson, 2014, Levinson, 2016, Noveck, 2018, Sauerland and Schumacher, 2016, Soroker et al., 2005). Substantial linguistic and neurocognitive research has focused on cases where pragmatic processing is most pronounced, that is, in non-literal meanings, including indirect speech, metaphors, irony and humour (Bambini et al., 2011, Bambini et al., 2019, Boux et al., 2022, Canal and Bambini, 2020, Coulson, 2008, Eviatar and Just, 2006), on the study of Gricean conversational implicatures (Benz and Gotzner, 2021, Degen and Tanenhaus, 2011, Feng et al., 2021, Gotzner et al., 2018, Hartshorne et al., 2015, Noveck and Posada, 2003, Zhan et al., 2017) or addressing social and pragmatic deficits in various clinical populations (Bambini et al., 2022, Baron-Cohen, 1988, Carotenuto et al., 2018, Deliens et al., 2018, Holtgraves and Giordano, 2017, Soroker et al., 2005). Further research has focused on the organisation and structure of conversations, which have yielded important insights on how human social interactions are organised in sequences (e.g., Kendrick et al., 2020, Levinson, 2013, Schegloff, 2007), where linguistic signs (words and sentences along with non-verbal communication, such as gestures) are used as a tool of communication to carry out linguistic actions, the so-called speech acts. Recent research has discovered novel brain signatures underlying pragmatic features of speech acts at the level of propositional content, action sequence structure, related commitments, and social aspects. The present paper focuses specifically on these recent advances concerning the neural processes of speech acts. I start by outlining standard linguistic-pragmatic theories along with a detailed description of the relevant pragmatic features that distinguish between speech act types. This is followed by a description of a neurocognitive model, the “Action Prediction Theory of Communicative Function”, which provides an explanation of the complex pragmatic processes involved in processing speech acts at the neurocognitive level. Next, the model is discussed in terms of recent advances regarding the long-standing debate in neuroscience about (i) when brain indexes of the linguistic-pragmatic information about communicative functions first occur and (ii) their cortical origins in mind and brain. Finally, I conclude with an outlook on what is needed in the future by highlighting the crucial importance of the mutual exchange of neurobiological approaches and linguistic-pragmatic theories to advance our understanding of the neural substrates of pragmatic knowledge regarding communicative functions in mind and brain.

## 2 Speech act theory: linguistic signs in action

Philosophy of language and linguistic pragmatics have provided extensive theoretical accounts of how linguistic utterances are used as a tool of communication to perform various actions in context (Alston, 1964, Austin, 1975, Ehlich, 2007, Ehlich, 2010, Fritz, 2013, Fritz and Hundsnurscher, 1994, Grice, 1975, Horn and Ward, 2008, Meibauer, 1999, Searle, 1969, Van Dijk, 1977, Wittgenstein, 1953). Defining words as tools that have different functions in their use was first advocated by Wittgenstein, who claimed that the “actions in which language is interwoven” are the result of the rules and context in which communication takes place, the so-called *language games* (Wittgenstein, 1953). This view became central to Austin’s (Austin, 1975) and Searle’s (Searle, 1969) speech act theory, where utterances were defined as *linguistic actions* (or speech acts) that not only serve to express information but also to perform specific actions through language, such as promises, requests or warnings. Each time a speech act is produced, three different acts are entailed: (1) The *locutionary act*, which is the propositional content of what has been said (“give me an apple”), (2) the *illocutionary act*, which are the goals and intentions behind the speaker’s utterance (“requesting an apple”) and (3) the *perlocutionary act*, the effect a linguistic action can have on the listener (“B gives an apple to A”). Following Austin’s original proposal (Austin, 1975), Searle (Searle, 1979) proposed five big classes of speech acts based on their illocutionary force. *Assertives* express things or facts in the external world (naming, stating); *directives* make the X partner (addressee) do something for the speaker (requesting, commanding); *expressives* describe the inner emotional state of the speaker (thanking, apologising); *commissives* commit the speaker to doing something in the future (promising, threatening); *declaratives* change the state of the world (baptise or arrest). Alternative taxonomies of illocutionary acts have been proposed (Ballmer and Brennstuhl, 2013, Van der Auwera, 1980, Zaefferer, 2001) and Wittgenstein emphasised the infinite variants of language games (Wittgenstein, 1953) stressing the difficulties of constructing an exhaustive catalogue of speech acts. Nevertheless, Searle’s taxonomy is a good starting point and is widely used for empirical research.

**Fig. 1:**
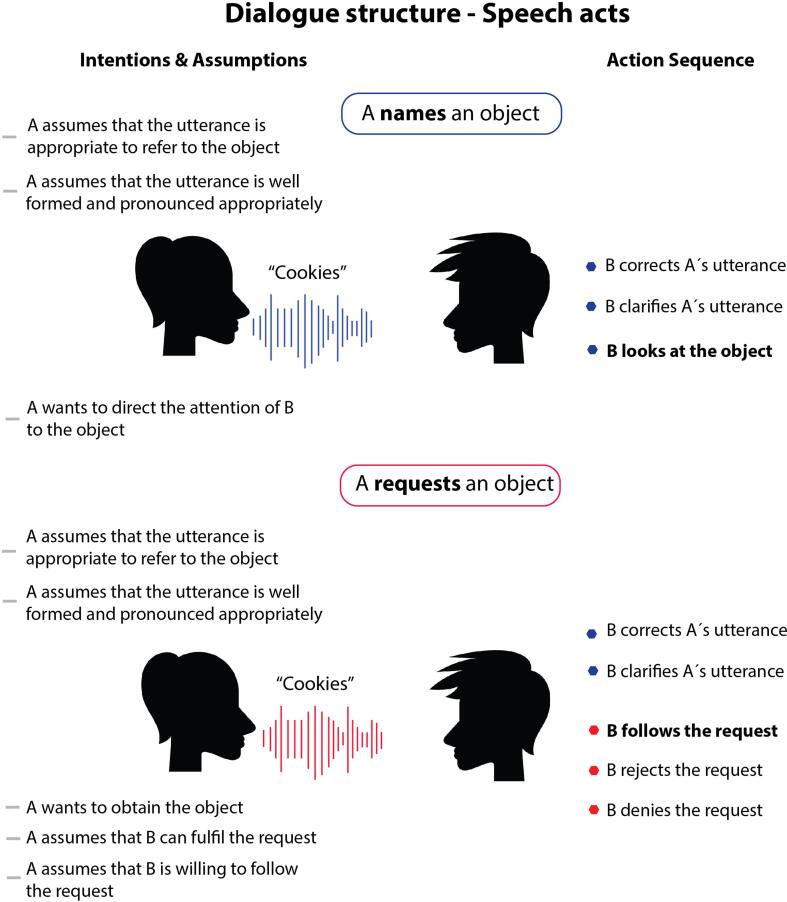
**Dialogue structure differences for the speech act of naming (top) and requesting (bottom).** Intentions and assumptions closely linked to the speaker’s intention are on the left and the action sequence structure, which describes the typical action of the communicative partner that follow the specific speech act, is on the right. Figure adapted from Egorova et al., 2013.

Since these philosophical and linguistic considerations described above, extensive work has been done on defining the essential features of the pragmatic functions of speech acts, dialogue structures, and other features of communication that are generally distinguishable at the level of linguistic signs, the actual actions that follow it and commitment structure (Alston, 1964, Clark, 1996, Ehlich, 2007, Ehlich, 2010, Fritz, 2013, Fritz and Hundsnurscher, 1994, Grice, 1975, Horn and Ward, 2008, Meibauer, 1999, Van Dijk, 1977). The following are essential:

i. *Propositional content*: the linguistic structures (words and sentences) with which a speech act is performed, i.e., the propositional content itself;
ii. *Communicative setting*: the non-linguistic aspects of the setting in which the utterances are embedded, including the physical environment in which the communication takes place and the objects present;
iii. *Action sequence structure*: the partner action responses preceding and following a given speech act, which are typically embedded in communication (Alston, 1964, Kasher, 1987);
iv. *Intentions and assumptions:* the specific assumptions and intentions to what the interlocutors commit to during communication (H. P. Grice, 1968, Hamblin, 1970, Kasher, 1987, Lewis, 1979, Walton and Krabbe, 1995), including shared knowledge between communicative partners (common ground, Stalnaker, 2002), aspects of which are sometimes called “theory of mind” (ToM);

Several of these linguistic-pragmatic features characterise the various speech act types differently. Consider, for example, the use of the utterance “cookies” (i) in a physical context (ii) to either name or request cookies and where the structure of the action sequence (iii) as well as the interlocutor's intentions and assumptions (iv) would vary according to the communicative function the utterance conveys. In a naming scenario, speaker A assumes that he or she is using the correct label to refer to the object (e.g., cookies and not cake), that the utterance is uttered and pronounced correctly (e.g., /ˈkʊkiz/, IPA transcription), and is thus understandable in all its components. This also includes the speaker's willingness to express it to the partner and the assumption that he or she might be interested in the item being referred to. The possible actions of listener B following the utterance are tied to these assumptions, where the options are either to correct the speaker's utterance (that the speaker meant cookies and not cake), clarify it (e.g., asking back what the speaker is referring to) or confirm (via verbal or non-verbal signal) having seen the object (Fig. 1 panel top). In a requesting scenario, speaker A’s assumptions include those in the naming scenario and add to them the assumption that the partner is willing and able to comply with the request and ultimately the speaker's desire to obtain the object. The actions following a request parallel those in the naming scenario, where listener B in response can clarify or correct the speaker’s utterance but also perform the requested action or reject or denies it by communicating that he or she is unable (e.g., there are no cookies left) or unwilling to carry it out (Fig. 1 panel bottom). Other specific pragmatic features for naming and requesting speech acts could be listed, yet the ones described here and shown in Fig. 1 are the most striking and useful to illustrate the main differences between these two speech acts. In short, at the pragmatic level the differences between the functions of naming and requesting rely on the action sequence structure (iii) and the intentions and assumptions (iv). Specifically, that request actions are characterized by additional assumptions and tied to the expectation of the partner's response of manipulating an object as compared to a naming action, which can have different implications for how these speech acts might be represented in the human brain (see next section).

## 3 Action prediction theory of communicative functions

A neuromechanistic model of communicative functions has been proposed, the so-called “Action Prediction Theory of Communicative Functions” (APC, Pulvermüller et al., 2014), which extends existing neurobiological models of language processing, which have mainly focused on the cognitive processes of linguistic structure such as phonological, morpho-syntactic and semantic processing (Damasio et al., 2004, Kemmerer et al., 2012, Pulvermüller and Fadiga, 2010, Tomasello et al., 2017, Tomasello et al., 2018). The APC model draws on the linguistic-pragmatic considerations described above and integrates insights from the neurobiology of language by offering precise predictions about the pragmatic features that distinguish between communicative functions at the neurocognitive level. The fundamental premise is that at the neural level, speech acts involve neural circuits by which speaker's assumptions and intentions along with the typical sequence of actions that follow it are processed. To illustrate how the different speech acts might be represented in the brain according to the model, let's consider the examples of naming and requesting given above.

When naming an object to direct the listeneŕs attention to the external object a core element is the semantic referential link between the word form and the object in the outside world. Thus, when understanding a naming situation, semantics-related regions shown to be involved in lexical-semantic processing, such as the inferior temporal regions or areas in the parietal-occipital lobe (Binder et al., 2009, Pulvermüller, 2013), are expected to be strongly active and involving only the left hemisphere. In contrast, understanding a verbal request may involve the motor action system, reflecting the expectation of the typical partner’s action of grasping an object and handing it to the speaker. This includes the mirror system and motor regions (Ortigue et al., 2010, Pulvermüller and Fadiga, 2010, Rizzolatti et al., 1996), specifically the motor regions that control the hand, as the object requested (“cookies”) is expected to be manipulated with the hand. Note that regions related to semantic processing (i.e., speech content) are also expected to be active in a requesting situation, but to a lesser extent than in a naming situation, as the speaker’s intention is to obtain the object. Additionally, due to differences in commitment structure between requesting and naming, in particular the fact that requesting is characterised by additional assumptions, whereby the speaker assumptions that the partner is willing and able to comply with the request, theory-of-mind (ToM) network - i.e. the right temporal junction or anterior cingulate regions, which have been shown to be involved in mentalising and social inferencing during communication (e.g., Van Overwalle and Baetens, 2009) - is also expected to be strongly activated. Overall, the idea is that speech acts are tied to their predictable sequences of actions, which are a crucial part of their meaning and therefore necessary for their understanding. In conversation analysis, these are typically referred to as “adjacency pairs”, where the speech acts and the response are interdependent (e.g., Schegloff, 2007). Here, however, the focus is on the entire set of possible action sequences that can follow a speech act and not just the typical one (e.g., question followed by an answer). Therefore, it is assumed that the entire set of expected (predicted) partner actions can be activated at the neural level from which speech acts derive their meaning. To emphasise this point again, the term 'prediction' here refers to the multiple alternative responses or predictable sequence of actions following a speech act (see Fig. 1), which may form an essential part of the mental representation in the brain at the cognitive level.

The APC model can also be employed as a test case for linguistic-pragmatic debates in speech act taxonomy (Searle, 1979). For instance, it has been claimed that Searle’s class of directives inappropriately includes questions. While the intention to “request verbal information” seems to function like requests (Searle, 1975, Searle and Vanderveken, 1985), other linguists have argued that an appropriate response to a question is an assertion, causing the speaker to update his or her information (i.e. common ground, Clark, 1996), functioning markedly differently from requesting an object (Groenendijk and Stokhof, 1997, Kiefer, 1980, Portner, 2004). The latter argument would define questions as being more like assertives with the key feature of directives being absent in question processing. If questions are directives and function like requests, the APC model would predict engagement of the articulatory-motor regions, reflecting the expectation of the partner’s typical action of uttering words to provide the desired information. However, if questions function as assertives, regions related to semantics should be active. Note that understanding questions, regardless of their similarity to a directive or assertive function, may additionally involve ToM regions, due to its richer commitment structure associated with the speaker's desire to receive the information that the partner might know and is willing to comply with the request, compared to a typical assertive speech act.

Given these considerations, neurocognitive experiments could be used to explore whether general brain signatures are at work for speech acts of the same category, thus (dis)confirming a speech act membership belonging to a category. It is agreed upon that linguistic pragmatic theories and issues should be critically addressed experimentally (Noveck & Reboul, 2008) and in recent decades a new stream of research in the areas of neuropragmatics has targeted how pragmatic processes in communication are instantiated in the mind and brain. Such research has great potential to inform linguistic-pragmatic theories and cognitive models of language processing (Bambini et al., 2011, Bara et al., 1997, Cutica et al., 2006, Gambi et al., 2015, Hagoort and Levinson, 2014, Levinson, 2016, Sauerland and Schumacher, 2016, Soroker et al., 2005).

## 4 Brain dynamics of speech act processing

A long-standing debate between linguists and cognitive scientists in experimental pragmatics is how early brain indexes of linguistic-pragmatic information about communicative functions occur. Upon perceiving a word like “cookies” in a request to obtain them, when would the speaker’s communicative intentions be processed? Very quickly, immediately after word onset, or only later, once phonological, semantic and/or morphosyntactic information has been processed?

**Fig. 2:**
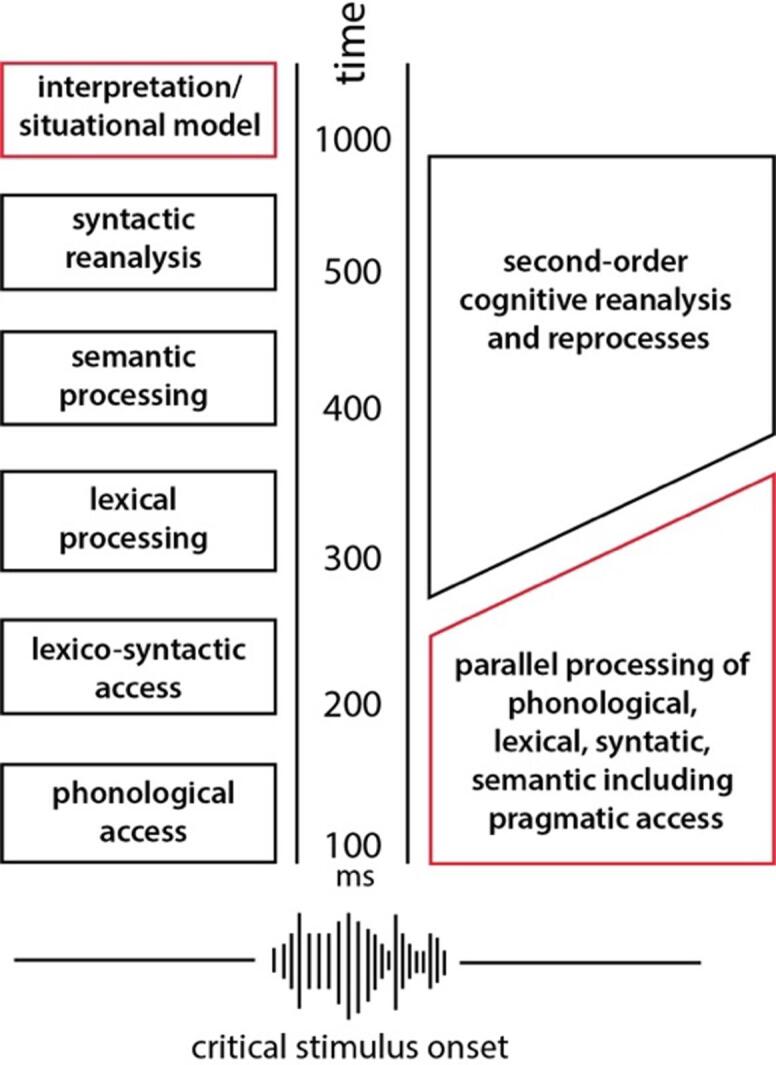
**Schematic representation of cascade/serial models and instant/parallel models of language processing.** The red highlighted box indicates where the two models would assume linguistic-pragmatic processing of speech acts during language understanding. Adapted from Pulvermüller et al., (2009).

Intuitively, one would assume that comprehension mechanisms during the perception of an utterance proceed in discrete steps, where phonetic/phonological information has to be processed before accessing higher-level semantic, syntactic and pragmatic information, which may also be retrieved in sequential steps. This view is consistent with most current psycholinguistic models of language comprehension, which advocate the serial processing of different linguistic representations in a cascade fashion. Upon hearing a linguistic utterance, the cascade comprehension timeline would start with processing phonological information followed by lexico-syntactic access and several stages of lexical and semantic analysis, and only at the end would pragmatic comprehension (i.e., interpretation) come into play (Fig. 2, boxes on left). Crucially, the delays between the different representations are in the range of 100 ms, suggesting that interpretation of the literal semantic meaning of an utterance does not occur until 400 ms after onset and that the processing of pragmatic information will not occur before 1000 ms (Friederici, 2002, Friederici, 2011). Other cascade models (Pickering and Garrod, 2004, Pickering and Garrod, 2013) advocate more flexible processing of the different linguistic levels, but the processing of pragmatic information (i.e., interpretation/situational model) at the final stage is common to these cascade models. In contrast, the so-called instant/parallel models advocate early and parallel processing of the different linguistic representations, where access to all representations occurs in parallel or nearly simultaneously (within 200 ms) during the perception and recognition processes (Fig. 2, boxes on right, Marslen-Wilson, 1987, Marslen-Wilson and Tyler, 1975, Pulvermüller et al., 2009, Shtyrov, 2010, Strijkers et al., 2017). The key research questions, therefore, are: Do pragmatic processes in speech act types occur early or late and do they occur in parallel with other linguistic information or in discrete steps?

To address these critical questions, a series of studies employing electroencephalography (EEG) investigated in the millisecond range when pragmatic information of speech acts is accessed during the understanding processes in written, spoken, prosodic and gestural contexts. Specifically, cases were examined where the propositional content (i) and the physical environment (ii) were identical but varied in terms of pragmatic differences in speakeŕs intentions (iii) (Boux et al., 2021, Coulson and Lovett, 2010, Egorova et al., 2013, Egorova et al., 2014, Gisladottir et al., 2015, Gisladottir et al., 2018, Tomasello et al., 2019, 2022, see the section “*Speech act theory: linguistic sign in context*”). Earlier studies used experimental set-ups, in which the same linguistic form, “flower”, was used to perform a naming (assertive) or requesting (directive) function in response to the context sentences “what are these called?” and “what can I get you?”, respectively (Egorova et al., 2013, Egorova et al., 2014). Surprisingly, when participants watched video tapes of two people interacting, therefore, taking an observer perspective, very fast neurophysiological responses were found at 150 ms after the critical word onset, with stronger activation for requesting than for naming (Egorova et al., 2013, Fig. 3 A). A follow-up study recording brain responses with magnetoencephalography (MEG) showed differences between the two speech acts even earlier, at 50–90 ms (Egorova et al., 2014). Although these studies demonstrate very early pragmatic processing, the predictive information provided by the context sentence prior to the critical word is somewhat problematic, as it may have triggered responses earlier than more natural, unpredictable communicative scenarios would.

**Fig. 3:**
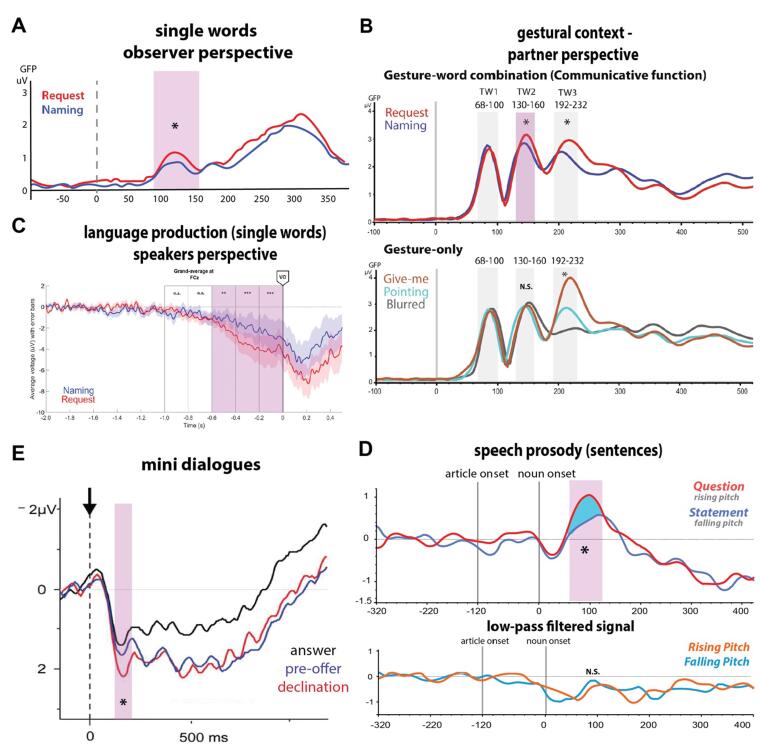
**Brain temporal dynamics of different speech act types.** A. ERP responses to request and naming scenarios expressed with single words from Egorova et al., 2013. B. Main ERP responses during request and naming understanding scenarios in a gestural context from Tomasello et al., 2019. C. Predictive brain activity prior to speaking in naming and request communicative scenario from Boux et al., 2021. D. Results of the brain responses of question and statement function conveyed by speech prosody, along with brain responses of low-pass filtered critical sentences from Tomasello et al., 2022. E. ERPs of the target utterance acting as a response to a question, a pre-offer to a statement or a declination of an offer from Gisladottir et al., 2015. The highlighted windows in magenta show where significant early neurophysiological differences of speech acts were detected.

The rapid pragmatic processing was confirmed in a recent EEG study also examining naming and request functions in an experimental design, in which speech act type and referential information were presented simultaneously (i.e. without prior information about the upcoming speech act). Moreover, the speech acts were addressed directly at the participants, so that the subjects took on the role of the partner (Tomasello et al., 2019). In particular, the same words were presented together with a pointing and give-me gestures having the function of naming or requesting objects (see e.g., Bates, 2014, Kelly, 2006). Interestingly, early and distinct brain responses were detected about 150 ms after their onset. In contrast, brain dynamics became evident much later when only information about speech act type (gestures presented alone) was available, that is, without referential semantic information (Tomasello et al., 2019, Fig. 3 B). These results support the notion of early processing of pragmatic information, but add that this only occurs when semantic information (speech content) is available, providing evidence for early and parallel processing of different linguistic information (Marslen-Wilson, 1987, Marslen-Wilson and Tyler, 1975, Pulvermüller et al., 2009, Shtyrov, 2010, Strijkers et al., 2017).

Brain indexes of speech act types have been shown to also appear before speaking during real-life interaction with an interlocutor (Boux et al., 2021). When naming or requesting an object from a partner, an ERP component resembling the readiness potential was shown to be sensitive to linguistic-pragmatic information prior to speech onset, and thus named “pragmatic prediction potential (PPP)” (for works in the semantic domain see e.g., Grisoni et al., 2021). Specifically, a negative-progressive response 600 ms before speaking was found to be more responsive for requesting than naming functions (Fig. 3 C). These results show that similar neural responses documented in speech act understanding are also involved prior to production. However, determining how early pragmatic processing occurs in production calls for additional research, as the slowly rising prediction potential and the lack of other variables (e.g., semantic) make it impossible to determine the temporal aspects of pragmatic processes.

Turning to other types of linguistic actions in other modalities, Tomasello et al. (2022) explored the brain correlates of question and statement functions conveyed by speech prosody and expressed with the same spoken sentence. In this study, Italian language sentences were used with different pitch contours (or fundamental frequency, F0), which are usually the only cues signalling either a statement (falling pitch) or a question (rising pitch) (e.g., Bolinger, 1978, Ohala, 1994). In line with previous studies, the results showed surprisingly instantaneous neurophysiological differences at 100 ms after the critical word differing in prosody. Whereas, in cases where there was no speech content and only the pitch contour was perceived (low-pass filtered sentences), in which subjects were still able to distinguish between speech act types, no comparable neurophysiological response differences were observed (Tomasello et al., 2022, Fig. 3 D). Consistent with a previous study (Tomasello et al., 2019), the findings indicate that the human brain is able to rapidly grasp the speaker’s intentions only when semantic information is available and perceived and demonstrate that this is also the case when prosody alone defines speech act types.

While all these findings show that speech act recognition is instantaneous, another study revealed differences in brain dynamics resulting from differences in dialogue structure (Gisladottir et al., 2015). This study examined mini-dialogues in which the same target-response utterance was preceded by context-specific sentences defining the speech act type. For instance, the sentence “I have a credit card” functioned as: an answer to the question “How are you going to pay for the ticket?”, a declination in response to the offer “I can lend you money for the ticket”, and a pre-offer in response to a statement “I don’t have any money to pay for the ticket”. Although early brain responses occurred at 200 ms for declination versus question responses, later neurophysiological differences were evident at 400 ms for pre-offer versus question responses (Gisladottir et al., 2015, Fig. 3 E). A follow-up study in which the same data were subjected to time–frequency analyses reported lower beta activity (12–20 Hz) for declination even before the target sentence, but no anticipatory activity was observed for pre-offer (Gisladottir et al., 2018). Differences in dialogue structure best explain these later-occurring neurophysiological differences. A statement like “I don’t have money to pay for the ticket” is usually not followed by any conventional partner action, in contrast to the question function, where a verbal response is expected. This makes a pre-offer unpredictable from the speaker’s utterance itself, but only when the target utterance is put into action, as the authors argued (Gisladottir et al., 2015). However, in the case of the pre-offer, much more is going on, since it involves a speech act change, from stating (assertive speech act type) to pre-offer (commissive type), where the speech act has to be inferred and reprocessed, causing additional pragmatic processing that may have led to the observed late neural processes. If the response to the statement “I don't have money to pay for the ticket” had only been an “okay”, confirming that the information had been received (i.e., the typical action sequence expected from a statement), faster processing may have been observed. However, this would make comparison with the other conditions difficult, as the target response would not be the same. Overall, it seems that speech acts are processed quickly, but when the action following it cannot be predicted and/or a speech act change occurs in conversation, later temporal activation can be observed. In another study examining non-conventional indirect requests, such as asking for a warmer soup via the utterance “this soup is cold” (which behaves similarly to the pre-offer condition above) compared to the same utterance functioning as a literal statement, early and late processing was observed in the second and fifth words, respectively (Coulson & Lovett, 2010). This evidence confirms that the processing of linguistic-pragmatic information begins early but can continue later during sentence processing. However, further work should look more closely to unravel the specific cognitive function of these early and late pragmatic processes and their underlying brain correlates.

In sum, EEG studies consistently show that brain correlates of speech act types occur rapidly in different modalities and experimental designs. Interestingly, however, quick pragmatic processing only occurs when pragmatic and semantic information is available during communication. These findings thus support neurocognitive parallel models (Fig. 2, right panel) that argue for early and parallel processing of different linguistic information, including pragmatic information. The rapid processing of linguistic actions is considered the key for the rapid exchange of turns between speakers and their partners, a well-known hallmark of efficient social-communicative interactions (Levinson, 2016).

## 5 Brain signatures underlying speech act types

Alongside discoveries about the rapid temporal dynamics of pragmatic processing, brain signatures for specific pragmatic features distinguishing various speech acts have been discovered by means of EEG/MEG source analyses (Boux et al., 2021, Egorova et al., 2014, Tomasello et al., 2019, Tomasello et al., 2022) and functional magnetic resonance imaging (fMRI, Bašnáková et al., 2015, Bašnáková et al., 2014, Egorova et al., 2016, Hellbernd and Sammler, 2018, Licea-Haquet et al., 2021, Van Ackeren et al., 2016, Van Ackeren et al., 2012, Fig. 4). A consistent finding is the immediate (∼150 ms) involvement of the hand motor cortex in understanding requests compared to naming functions (Egorova et al., 2013, Egorova et al., 2014, Tomasello et al., 2019), which is also supported by spatially accurate neuroimaging results (fMRI, Egorova et al., 2016), as well as in speech act production in interaction with a partner (Boux et al., 2021). The activation of the motor area for requests is in line with the predictions provided by the APC model (see section “*Action prediction model of communicative function*”). Requesting is intrinsically linked to the typical follow-up partner action of grasping an object and handing it to the speaker, which has been consistently documented to be reflected in the motor cortex activation during comprehension. In contrast, naming an object is not followed by any such action, rather, the focus is on the semantic referential information of the object in the outside world. Thus, in line with the APC model, the left angular gyrus in the parietal cortex, an area known to be active for referential semantic processing, was more strongly involved in naming than in requesting scenarios (Egorova et al., 2014, Egorova et al., 2016).

**Fig. 4:**
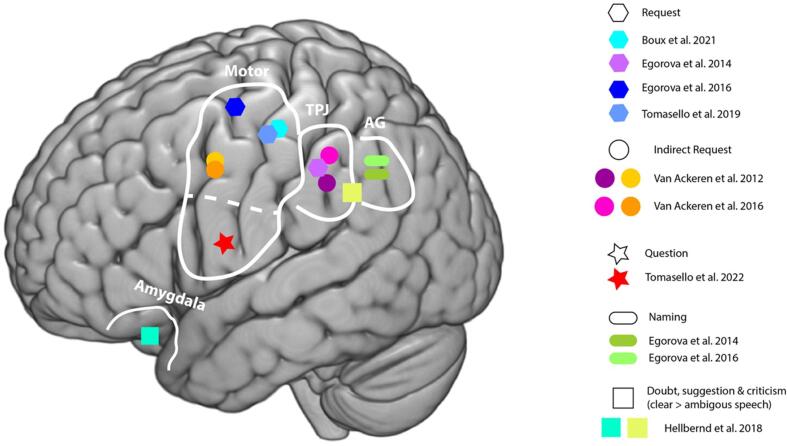
**Brain regions in studies investigating different speech act types**. Only activation of the left hemisphere is shown along with the pragmatic features relevant for action sequence and social and emotional aspects. Apart from the naming function, similar activations were also found in the right hemisphere. The data shown are from Boux et al., 2021, Egorova et al., 2014, Egorova et al., 2016, Hellbernd and Sammler, 2018, Tomasello et al., 2019, Tomasello et al., 2022, Van Ackeren et al., 2012, 2016. Motor regions; temporal parietal junction (TPJ); angular gyrus (AG); amygdala.

When it comes to understanding requests, not only is the follow-up partner action reflected in the mind and brain, but so its richer commitment structure, that entails additional assumptions as compared to naming, specifically the speaker's intention to obtain the desired object and the assumption that the partner can potentially fulfil the request and is willing to do so. In contrast, naming only commits the speaker to the correct referential labelling of the object in order to direct the partner’s attention to it (Fig. 1). The richer social-interactional knowledge inherent to requesting involved the bilateral temporal junction regions that belong to the core ToM network (Egorova et al., 2014), areas deemed crucial in processing the mental state of others, such as intentions, desires and beliefs (e.g., Van Overwalle & Baetens, 2009). However, MEG source analysis shows this activation at 200–300 ms, which is much later than the activation of motor areas at 50–90 ms after word onset (Egorova et al., 2014). This suggests that intentions and the action structure sequence are processed first, and other aspects of ToM may emerge later.

The ToM network seems to be strongly activated when understanding indirect requests relative to statements (Van Ackeren et al., 2012). Hearing the sentence “It is hot here” while being presented to a visual scene containing a closed window could be understood as an indirect request to open the window, whereas if a picture of the desert is presented, it expresses a statement. In line with previous studies described above (Boux et al., 2021, Egorova et al., 2014, Egorova et al., 2016, Tomasello et al., 2019), the results show that indirect requests involved both the action-motor regions related to its richer action knowledge, and the temporal junction and middle prefrontal regions, the cortical substrates of ToM. Intriguingly, functional interaction between these areas showed that motor region activation was driven by ToM regions and not by the core language areas (i.e., inferior frontal areas, Van Ackeren et al., 2016). The mentalising network activation (ToM area) has been interpreted as crucial for inferring pragmatic meaning, although whether it is related to indirectness or is part of the brain substrate for requesting, or a mix of both, is still an open matter. However, ToM regions, along with emotion areas, have been shown to be consistently being activated in processing indirect speech acts (e.g., direct vs indirect replies, Bašnáková et al., 2015, Bašnáková et al., 2014, Bendtz et al., 2022), yet different cognitive features of indirect speech acts compared to direct ones have been identified, making it difficult to relate the reported activations to a particular feature of indirectness (Boux et al., 2022).

Examination of brain substrates, in which speech prosodic cues conveying question and statement functions with rising and falling pitch, repsectively, showed instantaneous activation of the left articulatory motor regions (areas controlling lip/tongue movements) for questions 100 ms after the critical words differing in intonation (Tomasello et al., 2022). Note that in this study, the subjects' task was only to listen to the different sentences, and they were not instructed to perform any motor responses, so that the motor activation cannot be attributed to actual motor movements. Once again, the APC model comes into play as the best explanation for the specific motor locus revealed during question understanding in terms of the action sequence structure. A question is inextricably linked to the partner’s action of articulating words to provide the desired information, which is immediately reflected in the articulatory motor activity. These findings further illustrate that the action sequence typically following a speech act is part of its mental representation and relevant for its understanding. Furthermore, the results relate to the theoretical linguistic debate on the core features of questions and their appropriate classification into speech act categories by emphasising the predominance of an action (directive) component in question processing (for more detail see “*Brain data and theoretical implications – the case of question type*”). The presence of motor involvement for question functions was also found in a previous study that documented a ventral and dorsal auditory-motor pathway in the right hemisphere during single word processing (Sammler et al., 2015). Note that although these two studies reveal similarities in question processing, different hemispheric motor involvement was detected. One possible explanation is that Sammler et al., (2015) used single words and showed activation in the right hemisphere, Tomasello et al., (2022) employed spoken sentences, leading to a left hemisphere activation, defined as the core hemisphere for syntax processing (Friederici et al., 2000).

Other neuroimaging studies have shown involvement of the core ToM network as well as affect/emotion regions when understanding clear communicative functions (criticism, doubt and suggestions) conveyed by speech prosody relative to ambiguous ones (Hellbernd and Sammler, 2018, see Fig. 4), or in a speech act recognition performance contrasted to control conditions (Licea-Haquet et al., 2021). However, these studies used an active task (classification or recognition) involving two forced-choice tasks requiring a button press during the experiment. Active tasks are known to be associated with higher cognitive functions such as identification, attention, decision making and motor preparation, which may have covered relevant pragmatic fine-grained differences between the speech acts examined (e.g. see Schomers and Pulvermüller, 2016). Thus, a passive task would have provided more detailed insights into the neural substrates of the different speech acts investigated, possibly showing activation of motor regions for specific action-related speech acts, as consistently reported in other studies (Boux et al., 2021, Egorova et al., 2014, Egorova et al., 2016, Tomasello et al., 2019, Tomasello et al., 2022, Van Ackeren et al., 2012, Van Ackeren et al., 2016).

In sum, specific pragmatic features distinguishing between various speech act types are reflected differently in the human brain. In terms of the APC model, a consistent finding lies in the immediate activation of the motor cortex for action-related speech acts, which reflects the expectation that the partner will do something for the speaker (Boux et al., 2021, Egorova et al., 2016, Tomasello et al., 2019, Tomasello et al., 2022). The ToM network seems to be engaged for speech acts that are more socially complex (i.e., richer commitment structure) and enhanced in cases where linguistic actions are expressed indirectly. Here I also note that studies that use an active task are somewhat problematic due to the additional cognitive load associated with performing such a task. The findings reviewed here show that understanding speech acts crucially entails the knowledge of the typical partneŕs actions that follow them and that are part of their mental representation.

## 6 Brain data and theoretical implications – The case of question type

A theoretical linguistic debate addresses the core features of questions and their most appropriate classification into speech act groups. A study exploring the brain signatures of questions (Tomasello et al., 2022) has offered critical insights into this theoretical debate, in particular by showing how neurocognitive experiments and thus brain data can be useful in informing linguistic theories and issues.

Standard speech act theory defines questions as the intention to “request verbal information”, so questions are grouped with object-related requests into the category of directives (Searle, 1975, Searle and Vanderveken, 1985). Yet other linguists argue that questions should be distinguished from directive speech act types (Groenendijk and Stokhof, 1997, Kiefer, 1980, Portner, 2004), as an appropriate response to a question is an assertation, which is markedly different from requesting an object at various levels (e.g., updating of shared knowledge between interlocutors). This view would place questions halfway between directives and assertions and would be consistent with the notion that directives are not present in the processing of questions. If motor cortex activation is considered to reflect the action sequence following a request function (Boux et al., 2021, Egorova et al., 2016, Tomasello et al., 2019, Tomasello et al., 2022) and a brain signature of directive speech act types, it is reasonable to ask whether this type of activation is also present in other types of directives and thus in question types.

**Fig. 5:**
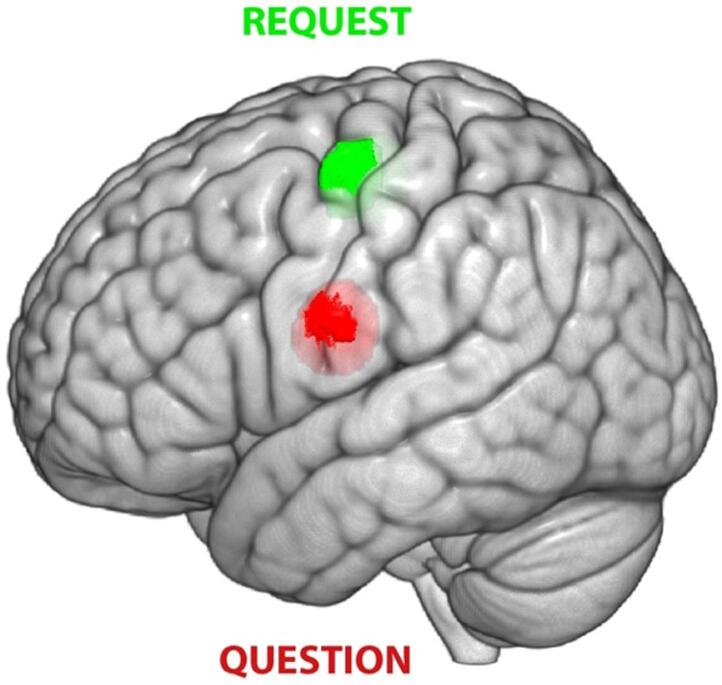
**Brain activation of request and question function within the motor cortex.** Requests: dorsal motor activation (in green; from Tomasello et al., 2019) in areas controlling hand motor activity. Questions: inferior motor activation (in red; from Tomasello et al., 2022) in the region involved in articulatory movement for spoken language.

Looking into the brain during question understanding has indeed revealed immediate activation of the motor regions, specifically the articulatory motor region, reflecting the typical action following a question (i.e., a verbal response, Tomasello et al., 2022, in red Fig. 5). This differs from requesting an object, where the follow-up action is performed with the hand and thus the hand motor cortex was demonstrably activated (Egorova et al., 2016, Tomasello et al., 2019, Van Ackeren et al., 2016, in green Fig. 5). These results indicate physiological similarities between questions and other forms of directives (requests to hand over objects) related to fast motor cortex activation and speaks for including questions in the category of directives, favouring Searle’s perspective. Moreover, the fine-grained motor cortex activation linked to the expected body part action movement (hand vs face representation), further supports the assumption that predictive knowledge are a crucially part of speech acts mental representation. Note that future studies need to replicate this evidence by exploring questions and requests in the same experiment, participants, and modalities. However, I’ve provided a clear example of how brain data can inform linguistic-pragmatic theories and issues, in this case speech act classification, by showing general brain signatures or physiological similarities that are indicative of similarities at the cognitive linguistic-pragmatic level.

## 7 Concluding remarks, future trends, and directions

Theoretical frameworks of linguistic pragmatics seek to describe and explain how language is used as a tool for communicating in context. Although such pragmatic frameworks have led to important theoretical considerations based on behavioural observations of language use and its consequences in conversations, they offer only indirect insights into the neural mechanisms at work in the human brain. Here I showed that neurocognitive studies allow direct observation of the spatio-temporal cognitive mechanisms of pragmatic processing of speech acts and can yield crucial insights into the complex system of language architecture and its function in social interaction. The crucial contribution of neurophysiological methods (EEG/MEG) has made it possible to study the brain dynamics underlying pragmatic information millisecond by millisecond, providing converging evidence for the ultra-rapid processing of pragmatic information occurring in parallel with other linguistic information (i.e., semantic), thus supporting parallel models of language processing (right-hand side, Fig. 2).

Source analysis (EEG/MEG) and brain imaging studies (fMRI) enabled the exploration of the cortical brain regions underlying speech act processing, which led to an interesting side effect: the discovery of specific brain signatures indicative of the processing of specific pragmatic features related to different speech act types. Consistent evidence includes the immediate activation (∼150 ms) of cortical motor regions related to the partner’s expected action following directive speech act type. For example, the hand motor cortex was found to be consistently activated during basic object-related verbal requests representing the partner's expectation of object manipulation (Boux et al., 2021, Egorova et al., 2016, Tomasello et al., 2019, Van Ackeren et al., 2016), and the articulatory-motor region is likewise activated for question function, mirroring the preparation of a vocal response (Tomasello et al., 2022). Overall, these results provide initial evidence that there are specific brain signatures indicating that the expectation of partner action following a speech act is part of the mental representation.

Although neuroscientific methods allow for the exploration of neural mechanisms underlying pragmatic processing and social interaction, linguistic-pragmatic theories are equally useful and crucial in informing neurocognitive experimental studies and their set-ups to investigate the specificities of different speech act types. Based on this foundation, a neuromechanistic, action prediction model of communicative functions has been proposed that can provide a range of possible predictions about the brain correlates of different speech acts. Such predictions can be validated by looking at the brain and, in turn, findings deriving from empirical neuro-cognitive approaches can inform unresolved debates in linguistic theory in a mutually fruitful exchange.

An important conclusive note is that research into language use and communicative functions and their neural correlate in the human brain is still in its infancy. Although these initial results support the proposed APC model, further studies of speech act processing in different modalities and experimental settings are needed to further test the validity of the model. Further studies could, for example, investigate whether patients with lesions in the motor cortex are impaired in understanding action-related speech acts such as requests and questions. This would support the thesis that the predictive action sequence that follows a speech act is functionally relevant for its understanding. Besides, only a few speech acts (mostly in the directive and assertive category) have been researched from the perspective of neuroscience, less attention has been given, for instance, to expressive or commissive acts. Moreover, because communication requires two or more interacting persons (Holler and Levinson, 2019, Levinson, 1983), which has been defined as *joint actions* (Clark, 1996), there is the need to replicate and confirm the findings described in neurocognitive studies in ways that more closely resemble real-life interactions. Since laboratory experiments are often far from reality, in which experimental variables known to influence natural conversation have not yet been sufficiently explored (e.g., common ground, joint attention, eye gaze etc.). Recent research has attempted to achieve such an approximation, for example by including a “confederate” who enacts dialogue participation (see e.g., Bögels et al., 2015, Boux et al., 2021, Rueschemeyer et al., 2015) or the use of dual recordings or so-called hyper-scanning methods, where two interlocutors (a speaker and a listener) are simultaneously scanned during social interaction (see for a review Czeszumski et al., 2020, Kuhlen et al., 2015). New insights in speech act understanding and production during interaction can be tackled by using such methods, specifically answering also critical questions about neural synchronisation. Recently, novel, sophisticated computational methods have also been proposed to align data points with stimulus presentation when studying continuous natural speech in context (Schilling et al., 2021); such a method could be adopted for the study of pragmatic processing of speech and interaction or even be combined with the more real-life experimental settings mentioned above.

Although much work is still needed to further advance our understanding of the complex system of language architecture and its function in social interactions, all the research discussed here shows promising ways to investigate the brain mechanisms involved in communication. It further shows that linguistic-pragmatic theories are powerful tools for guiding neurocognitive pragmatic models (i.e., APC) and experimental research, and that their findings can, in turn, refine theories and ultimately lead to a better understanding of how communicative functions are processed at the level of linguistic actions, mental processes and neural circuits.

## Declaration of Competing Interest

The author declare that he has no known competing financial interests or personal relationships that could have appeared to influence the work reported in this paper.
